# Microtubules as Sub-Cellular Memristors

**DOI:** 10.1038/s41598-020-58820-y

**Published:** 2020-02-07

**Authors:** Jack A. Tuszynski, Douglas Friesen, Holly Freedman, Valery I. Sbitnev, Hyongsuk Kim, Iara Santelices, Aarat P. Kalra, Sahil D. Patel, Karthik Shankar, Leon O. Chua

**Affiliations:** 1 Department of Oncology, University of Alberta, Cross Cancer Institute, Edmonton, AB Canada T6G 1Z2; 2 Department of Physics, University of Alberta, Edmonton, AB Canada T6G 2E1; 3 DIMEAS, Politecnico di Torino 10129 Turin, Italy; 4 Li Ka Shing Institute of Applied Virology, University of Alberta, Edmonton, AB Canada T6G 2E1; 5 St. Petersburg B. P. Konstantinov Nuclear Physics Institute, NRC Kurchatov Institute, Gatchina, Leningrad district, 188350 Russian Federation; 6 Department of Electrical Engineering and Computer Sciences, University of California, Berkeley, CA 94720 USA; 7 Division of Electronics Engineering, Chonbuk National University, Jeonju, Jeonbuk 561-756 South Korea; 8 Department of Electrical & Computer Engineering, University of Alberta, Edmonton, AB Canada T6G 1H9

**Keywords:** Bionanoelectronics, Supramolecular assembly

## Abstract

*Memristors* represent the fourth electrical circuit element complementing resistors, capacitors and inductors. Hallmarks of memristive behavior include pinched and frequency-dependent I–V hysteresis loops and most importantly a functional dependence of the magnetic flux passing through an ideal memristor on its electrical charge. Microtubules (MTs), cylindrical protein polymers composed of tubulin dimers are key components of the cytoskeleton. They have been shown to increase solution’s ionic conductance and re-orient in the presence of electric fields. It has been hypothesized that MTs also possess intrinsic capacitive and inductive properties, leading to transistor-like behavior. Here, we show a theoretical basis and experimental support for the assertion that MTs under specific circumstances behave consistently with the definition of a memristor. Their biophysical properties lead to pinched hysteretic current–voltage dependence as well a classic dependence of magnetic flux on electric charge. Based on the information about the structure of MTs we provide an estimate of their memristance. We discuss its significance for biology, especially neuroscience, and potential for nanotechnology applications.

## Introduction

## Memristors

The term memristor is the contraction of memory and resistor and it was first proposed in 1971 as the fourth element of the electric circuits^1^. A memristor is defined as a two-terminal passive circuit element that provides a functional relation between electric charge and magnetic flux^1,2^. The first physical realization of a memristor was achieved in 2008^2,3^ and it has held a promise of nanoelectronics beyond Moore’s law^4^, although this realization has been both difficult and controversial^5^. One of the possible breakthrough applications of memristors is neuromorphic computing^6^. *Memristance* refers to a property of the memristor that is analogous to resistance but it also depends on the history of applied voltage or injected current, unlike in other electrical circuit elements. When the electrical charge flows in one direction, the resistance of some memristors increases while it decreases when the charge flows in the opposite direction or vice versa. If the applied voltage is turned off, the memristor retains the last resistance value that it exhibited. This history dependence of memristance is expressed via a self-crossing or pinched I–V loop, which is frequency dependent^3,6^, and whose lobe area tends to zero as the frequency tends to infinity.

A memristor is said to be *charge-controlled* if the relation between flux *φ* and charge *q* is: *φ* = *φ (q*). Conversely, it is said to be *flux-controlled* if *q* = *q(*φ*)*. The voltage *v* of a charge-controlled memristor obeys a linear relationship with the current *i(t)* representing a charge-dependent Ohm’s law such that:

1 $${v}{(}{t}{)}={M}{(}{q}{)}\,{i}{(}{t}{)}$$

where memristance is defined as:

2 $${M}{(}{q}{)}={d}\varphi {(}{q}{)}/{dq}$$

and it has the units of resistance, namely ohms. For a flux-controlled memristor: *i(t)* = *G(φ) v(t)* where the proportionality coefficient *G(φ)* = *dq/dφ* is called *memductance* (acronym for memory conductance) and is the inverse of memristance.

The ideal memristor^1^ has been generalized to any electrical circuit device exhibiting a pinched hysteresis loop^7^. Such generalized memristors have been identified in numerous naturally occurring systems, e.g. potassium and calcium ion channels in the Hodgkin–Huxley nerve membrane circuit model^8,9^, the aplysia habituation neuron in Kandel’s research on memory^10,11^, silk proteins^12^, organic memristors^13^ and conducting structures in plants^14–16^. The connection between memristors and neuronal synapses^17^ can potentially shed light on the enigma of memory generation, erasure and retention in the human brain. In this context, a molecular model of memory encoding has been based on phosphorylation of neuronal MTs by calcium calmodulin kinase enzyme (CaMKII)^18^. This provides indirect indication that MTs may function as nano-scale sub-cellular memristors with an enormous potential for storage of large amounts of biologically-relevant information. Their involvement in many biological functions, especially in cell morphology, mitosis, intracellular transport and neuronal migration makes them important biological structures whose memristive properties would provide an extraordinary range of possibilities in the context of cell biology and neuroscience and also offer a great potential for hybrid nano-biotechnological advances using a combination of protein-based and synthetic components.

## Microtubules

The cytoskeleton of eukaryotic cells contains three main types of protein filaments, namely: MTs, actin filaments (AFs) and intermediate filaments. In addition to providing the necessary mechanical rigidity for cell morphology and localized force generation capabilities due to their polymerization dynamics, these protein polymers participate in a multitude of key biological functions including cell division, cell motility and intracellular transport. Additionally, MTs and other cytoskeletal filaments in neurons have been hypothesized to store molecular bits of information that can build up memory at a sub-cellular level. They have also been proposed to transmit electrical signals in neuronal cells^16–20^. Conducting properties of AFs and MTs have been experimentally and computationally investigated yielding important insights into their remarkable behavior, which is discussed below. Importantly in this connection, both AFs and especially MTs possess highly electrically-charged surfaces, which enable them to conduct electrical signals *via* ionic cable-like transmission process^21,22^. It is important to note that MTs are very abundant in neurons where they form parallel bundles interconnected by MAPs (MT-associated proteins) resembling parallel processing computational architecture. It is, therefore, unsurprising to find that the key components of this intricate subcellular architecture, namely MTs, are endowed with special electrical conduction properties.

MTs have been experimentally demonstrated to respond to externally-applied electric fields *in vitro* exhibiting alignment and drift effects along field lines^23–25^. However, electric conductivity determination for biopolymers has been very challenging because of both the structural non-uniformity and instability of these polymers and the need to maintain the samples in liquid solution. Moreover, it is also important to note that these biological polymers are very sensitive to environmental factors, e.g. ambient temperature, pH, and buffer composition, especially its ionic strength. A number of experiments have been performed attempting to circumvent these difficulties involving either the intrinsic^26–28^ or ionic^29,30^ MT conductivities. Direct measurements of ionic electrical conductivity along the MT axis placed in buffer solution in micro-channels established an upper limit on MT conductivity of 90 S/m^29^ and using an electro-orientational methodology^30^ resulted in an MT conductivity estimate of approximately150 mS/m for individual microtubules and a much lower value of approximately 90 mS/m for MTs in the presence of subtilisin (which cleaves C-terminal tails from tubulin reducing 40% of the net charge and hence MT conductivity). This suggests that positive counter-ions partially condense around the negatively-charged MT surface, including the negatively charged C-termini. The mobile counterions attracted to, but not condensed onto the solvent-exposed surface of MTs, appear to be the main contributors to the observed high conductance of MTs with a value approximately 15-fold greater than the solution’s conductivity (9.7 mS/m) in which they were placed in the conducted experiments. More recently, Sahu *et al*.^31^ tried to measure electrical conductivity due to counter-ions flowing along the outer surface of MTs. They reported the results from their four-probe measurements of both DC and AC conductive properties. The values of DC conductivity of MTs, found using a 200 nm gap, were reported to be in a very broad range from 10^−1^ to 10^2^ S/m. In fact, they found MTs at particular frequency values to become almost 1000-fold more conductive than their DC estimates, reportedly showing surprisingly high values for MT conductivities between 10^3^ and up to 10^5 ^S/m^31^. These effects were interpreted as being due to MT’s ballistic conductivity property. It was also claimed by these authors but not proven that the high conductivity at specific frequency ranges arises from the water content inside the MT lumen^32^. Moreover, Santelices *et al*.^32^ published precise measurements of the AC conductance of MTs in electrolytic solutions and compared them to analogous solutions containing unpolymerized tubulin, at various protein concentrations using a nanofabricated microelectrode-geometry system. Their results show that MTs at a 212 nM tubulin concentration in BRB4 buffer raised the solution’s conductance by 23% at 100 kHz. This effect scaled directly with the tubulin concentration in solution. However, a peak in the conductance spectrum positioned at around f = 110 kHz was observed to be concentration –independent while its amplitude decreased linearly with tubulin concentration. On the other hand, free tubulin was observed to have an opposite effect by decreasing the solution’s conductance by 5% at 100 kHz under identical conditions. Interpreting these measurements in terms of the number of MTs and approximating their electrical behavior as resistors networks acting in parallel surrounded by a lower conductance solution, it can be estimated that the single MT conductance is approximately 20 S/m. This can be compared to an approximate value of 10 mS/m estimated for the buffer and it indicates that MTs, under certain experimental conditions, exhibit unusually high electric conductivities, being roughly 1000-fold greater than those of the buffer solution. Further experimentation using a parallel-plate capacitor and physiologically-relevant concentrations of tubulin showed that MTs increased solution capacitance at cellular concentrations unlike free tubulin (A. Kalra *et al*., arXiv preprint arXiv:1905.02865, 2019). These data interpolated to a single 20 μm-long MT indicates the value of its capacitance as *C* = 3 pF which is comparable to the earlier computational predictions^33–37^. Some discrepancy between these results may be due to a significant reduction in the value of the dielectric constant of the solution near the protein surface, a fact not included in these previous computational estimates^33–37^.

**Figure 1:**
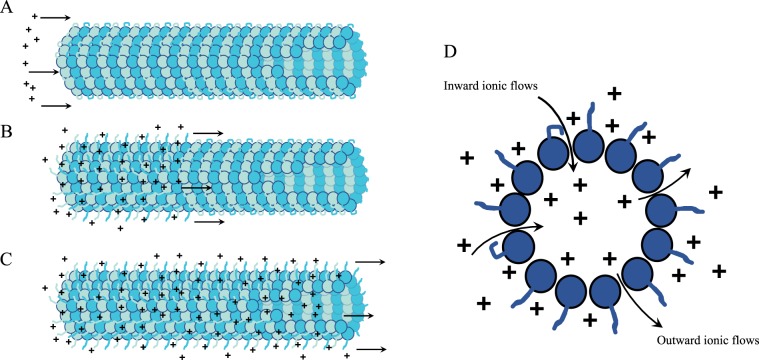
Schematics displaying ionic movement along an MT. Conformational changes in C-termini on the MT surface both alter ionic flows and respond to them, creating a non-linear transmission line. (**A**–**C**) display charge transport along the MT length and outside it. (**D**) displays charge transport across the MT surface into its lumen.

Based on the above overview of the reported effects of MTs on the conducting properties of solutions containing MTs, it can be concluded that MTs act as conducting cables for charge transport, showing increased conductivity compared to the solution itself as well as possessing electric capacitance that is due to counter-ion condensation and the formation of a charge-separation double layer involving negative MT surface charges and positive counter-ions. It was further hypothesized previously that MTs may also have intrinsic inductance due to the possibility of solenoidal flow of ionic charges, which put together with other observed properties and a non-linear capacitance due to highly limited number of counter-ions they can attract, leads to a transistor-like behavior with the observed injected current amplification^33,38^. Below, we argue that in addition to the already demonstrated unusual electrical conduction properties described above, MTs also behave as nano-scale sub-cellular memristive devices. In Fig. 1 we show how the effects of ionic charge propagation along an MT affect the conformations of the negatively-charged C-termini and also how these ionic flows may involve penetration into the MT lumen. These effects will be discussed below in the paper in connection with memristive behavior or MTs.

## Microtubules as Memristors

It is worth noting that approximately 50% of the net negative charge of a tubulin dimer resides in C-terminal protrusions that arise from the protein surface exposed to the bulk solution^30^. These C-terminal ‘tails’ have a large percentage of Asp and Glu amino acids, and have been computationally simulated^35^ showing that they likely exist in two major conformational states: (a) a flexible conformational state pointing away from the MT surface towards bulk solution, which indicates a major role of thermal fluctuations, or (b) a more stable state in which the C-termini bind electrostatically to the MT surface at tubulin areas with a local positive electrostatic charge. The two conformations are separated by a potential barrier that can be overcome by a local electric potential fluctuation. Each tubulin dimer has two C-termini that may thus extend outward toward the bulk solution or bend and bind to the MT surface. The state of the C-termini was modeled to modulate the flow of the solution’s ions radially across the MT cylinder in and out of the lumen, through so-called nanopores, which are present between the two adjacent MT protofilaments and spaced every 4 nm. A schematic of ionic flow along the outer surface of an MT has been shown in Fig. 1A–C. Counter-ion flows around the surface of an MT and long its axis are, therefore, dependent on such factors as local pH values, and on the local ionic concentration in the MT vicinity, as well as on the state of the MT’s C-termini making it a dynamic and possibly nonlinear system. Therefore, the effective radius of the MT structure depends on the state of the C-termini. A lowered concentration of counter-ions will cause a collapse of C-termini on the surface of a MT where patches of positive charges were found to electrostatically attract their negative charges^35^. It important to note that this effect is similar to the situation arising with current flows along memristors, which affect the state of the memristor^7^.

It has been described elsewhere^39^ that a memristor is an electrical analogue of a flexible pipe that changes its diameter with the amount and direction of fluid that flows through it. If the fluid flows through this pipe in one direction, it expands (becoming less resistive to fluid flow). When the fluid flows in the opposite direction, the pipe shrinks (becoming more resistive to fluid flow). Furthermore, the memristor “remembers” its diameter when the fluid last went through. When the fluid flow is turned off, the pipe diameter “freezes” until such time when the fluid flow is turned back on. The ability to indefinitely store resistance values means that a memristor can be used as a nonvolatile memory. This is in fact what the C-termini of an MT represent due to their conformational changes and the ability to either expand or contract radially as a result of the increased or decreased presence of counter-ion concentrations in their vicinity. Since the counter-ions act as electric charge carriers in the ionic currents facilitated by MTs functioning as nonlinear cables, the variable-diameter pipe analogy appears to be suitable for the description of MT conductivity.

Computational support for this conjecture comes from Freedman *et al*.^40^, who report simulations of the ionic currents through microtubule nanopores and the lumen in the presence of coupled C-termini dynamics. In this model, Freedman *et al*.^40^ use the Grand Canonical Monte Carlo /Brownian Dynamics (GCMC/BD) methodology to study ionic conductance along the lumen, which is affected by fluxes through the nanopores when an external potential is applied. Figure 1D schematically illustrates such ionic movement through nanopores into and out of the MT lumen.

These simulations revealed specific nanopore conductances and selectivity for an ionic species type. At positive voltages, protein charges increase total conductance by a factor of 7 and cation conductance by a factor of 15. At positive voltages, C-termini increase the total conductance by 12% and cation conductance by 11%, but there has been little effect found on anions (which are gated at the entrance). While the simulations of Freedman *et al*.^40^ did not explicitly show the existence of a pinched hysteresis loop in the ionic conductivity of MTs, this can be derived and analyzed from the data presented in this paper.

**Figure 2:**
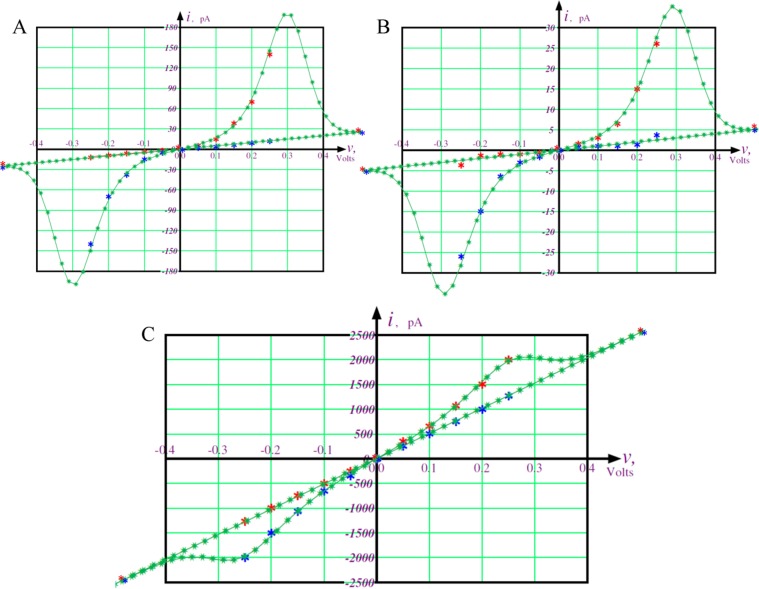
Diagrams showing the effects of nanopores in the current–voltage characteristics of microtubules resulting from the calculations based on the simulations in ref. ^40^. Blue asterisks depict the same dependence as red asterisks but with inverted currents and voltages. Fitted pinched hysteresis loops are shown with green asterisks. (**A**) Current-voltage relation from GCMC/BD simulation for conductance of anions through the type I pore. (**B**) Current-voltage relation from GCMC/BD simulation for conductance of cations through the type II pore. (**C**) Current-voltage relation from GCMC/BD simulation for conductance of anions through the type II pore.

In Fig. 2 we show the key findings regarding the current-voltage (I–V) characteristics for the two types of nanopores present in MTs and for anions and cations based on further analysis of the results of the computer simulations. Below we describe how the curves in Fig. 2 have been obtained mathematically.

The existence of a pinched hysteresis loop results from the I–V characteristics and it requires an inversion operation, which is straightforward to demonstrate. Namely, if *I* = *G*(*φ*)*V* is a solution describing the I–V characteristic for a microtubule, then (−*I*) = *G*(*φ*)(−*V*) is an odd-symmetric solution of that dependence as well. This relies on the definition of memductance given above as *G*(*φ*) = *dq*(*φ*)/*dφ*. If we reverse the signs of the charge *q* and the flux *φ* then memductance *G*(*φ*) does not change the sign. However, both the current *I* = *dq*/*dt* and the voltage *V* = *dφ*/*dt* do change their signs. Hence, the flux-dependent Ohm’s law *I* = *G*(*φ*)*V* does not change when changing the signs of *I* and *V* to the opposite ones. Consequently, it follows that we can find the dependence of *I* versus *V* for the negative values from that obtained by Freedman *et al*.^40^ by inverting the signs of *I* and *V*. Next, we follow the ideas stated in Chua *et al*.^7^. The problem is to find such a *G*(*φ*) for which the pinched hysteresis loop can be reproduced when *V* is described by a sinusoidal function.

It is important to note that the data represented by the red and blue asterisks in Fig. 2 intersect at the origin. These intersections have different slopes for the two curves representing the data obtained from the simulations of Freedman *et al*.^40^. We assume that these data result in the formation of pinched hysteresis loops^7^. With this in mind, we judiciously choose the following dependence of *q* versus *φ*

3 $${q}(\varphi )={{G}}_{0}\{\varphi +{\alpha }\varphi \,\tanh \,[(\varphi -{\varphi }_{0})/{\varphi }^{\ast }]\}$$

here, the four parameters, *G*_0_, *α*, φ*, and *φ*_0_, are adjustable. In particular, the parameters *G*_0_ and *G*_1_ = (1 + *α*) *G*_0_ are memductances at *φ* = *φ*_0_ and at *φ* ≫ *φ*_0_, respectively. Differentiating the above equation with respect to time *t*, we obtain^29^.

4 $${I}={dq}/{dt}=[{dq}(\varphi )/{d}\varphi ]({d}\varphi /{dt})={G}(\varphi ){v}$$

where

5 $${G}(\varphi )={{G}}_{0}\{1+\,{\rm{sech}} \,{[(\varphi -{\varphi }_{0})/{\varphi }^{\ast }]}^{2}\}$$

is the memductance at *φ*, and has the unit of siemens (S). Applying a sinusoidal voltage source given by the following relationship

6 $${v}({t})={A}\,\sin ({\omega }{t})\,{\rm{for}}\,{t} > 0\,{\rm{and}}\,{v}({t})=0\,{\rm{for}}\,{t} < 0$$

leads to the flux

7 $$\varphi ({t})={\int }_{0}^{{t}}{A}\,\sin ({\omega }{t}{^{\prime} }){d}{t}{^{\prime} }=({A}/{\omega })[1-\cos ({\omega }{t})]$$

For the sake of simplicity, we set the arbitrary parameters *A* and *ω* as *A* = 1 and *ω* = 1. The parameters *G*_0_, *α*, *φ*_0_ and φ* were adjusted in such a manner that the dependence of *i* versus *v* provides the best fit to the data shown in Fig. 2.

The non-zero parameter *φ*_0_ in the pinched hysteresis loops shown in Fig. 2 determines concavity of the upper right-side curve covering the red asterisks and the lower left-side one covering the blue asterisks. This parameter determines the asymmetry of the dependence of *q* versus *φ* with respect to the origin. It is clearly seen to have a negative value *q*_0_ at zero flux, *φ* = 0. This is in accordance with Freedman *et al*.^40^ and the previously described electrostatic characteristics of MTs, which have a large uncompensated negative charge to which *q*_0_ corresponds, thereby supporting our fitting procedure.

Note that the value of M of a memristor depends on the charge (i.e., the time integral of current), and not on the current itself. In fact, if a DC current, *i*(*t*), is applied across a memristor, the memristance will not have a constant value, but will vary with time. This is because the memristance *M*(*q*) = *df*(*q*)/*dq* is not a function of i, but rather it is a function of the time integral of *i*(*t*), namely, the charge *q*, where *f*(*q*) is the slope of the flux versus charge, a characteristic curve defining the memristor. After the current drops to zero at *t* = *T*, the memristance retains its last value *M*(*T*). In other words, the memristor remembers the latest value of *M*(*t*) until the current drops to zero. This property is directly responsible for the memory of the device. It is important to understand that what is remembered is the value of memristance, and not the value of the voltage, or current. Hence memristance represents the “memory” property of the device.

The question then arises regarding an estimate of the memristance of a single MT based on our understanding of its electrical conductivity properties. Using earlier analyses of MT conductivity as an effective RLC network forming an ionic conductivity cable^36,37,40^, the following estimates have been made for a single ring of an MT, which is 8 nm long. Its capacitance was found as *C* = 6.6 × 10^−4^ *pF*, its resistance perpendicular to the cylinder axis as *R*_2_ = 1.2 MΩ and its inductance as *L* = 30 pH, assuming the presence of solenoidal ionic currents winding tightly around and along its axis. Recall that memristance is the partial derivative of the magnetic flux with respect to electric charge. Magnetic flux, *φ*, is proportional to the magnetic induction and the cross-sectional area, *A*. Inductance is given by the formula: *L* = *μN*^2^*A*/*l* where *N* is the number of virtual coils wrapped around the surface of an MT and *N* = *d*/*rh* with *d* denoting the length of a dimer (8 nm) and *rh* the radius of hydration of an ion (0.36 nm). For the tightest possible winding around the cylinder N_max_ = 20 while for the least tight only 1 virtual ionic wire wraps around the entire MT, so N_min_ = 10^−3^. Using the formula for magnetic induction *B* = *μNI*/*l* and substituting for *L* from above we readily find that *φ* = *LI*/*N*. As determined in earlier MT conductivity experiments, typical current values along an MT are on the order of 1 pA^34^. We now hypothesize, based on the previous arguments, that memristance is due to the effects of C-termini undergoing conformational changes resulting from the ionic current flows. The two conformational states of the C-termini of MTs, namely outstretched and folded, based on the dimensions of the peptide chains involved, may differ by as much as 4 nm (but no more than that due to the size of these peptide structures), which would affect the effective radius of an MT just like a variable diameter pipe used as a metaphor above. Hence, a relative change in the inductance of an MT is estimated to be Δ*L*/*L* = 2Δ*R*/*R* = 0.6. Therefore, for a single ring of an MT, an associated change in inductance, Δ*L*, is expected to be in the range of 20 pH. This is expected to result from a change in the amount of net charge on the C-termini of approximately 5 to 6 *e*, hence Δ*Q* = 10^−18^ C. With these values and the fact that N = *d*/*rh* = 20, we obtain the memristance of a single MT ring as *M*_0_ = Δ*LI*/Δ*QN* ranging between 10^−3^ Ω and 20 Ω. Since an average MT is typically 10 µm long, M = *nM*_0_ where n is the number of rings *n* = l/*d* = 1250 and hence the total memristance for such an MT, *M*, is expected to range between 1 Ω and 20 kΩ.

This is a very small value for a single MT, whose conductance is expected to be on the order of 20 S/m^32^, hence resistance is expected to be in the range of 1 GΩ completely overshadowing the memristive contribution. Conversely, a very large memductance means that this mode of ionic conduction around a MT, in a solenoidal fashion involving the dynamics of C-termini represents a high conductivity “cable” with a special memristive property. This does not mean that memristive properties of MTs are insignificant but that they are related to a special mode of ionic conduction related to solenoidal currents affected by C-termini conformational states. We, therefore, hypothesize, that these solenoidal ionic currents require special initial conditions to be generated, i.e. low intensities of the electric fields generating them in order to maintain tight contact of ionic flows around MTs and an orientation of these electric fields at an angle to the MT axis, which is close to but not exactly perpendicular in order to lead to tight winding of the solenoidal flows that result. Otherwise, it is expected that ionic flows involving MTs would either proceed linearly along the MT axis, occur perpendicularly to it and cross the MT walls through its nanopores, or finally, scatter and diffuse off the MTs representing obstacles to ionic flows.

It should be mentioned in the context of biological applications that the memristive behavior of MTs could have remarkable applications in biology, especially as a memory-storing device. This will be discussed in more detail in the Conclusions section but it suffices to say that the direction of the solenoidal currents could be controlled by C-termini phosphorylation or post-translational modifications, both of which are known effects in cell biology and could explain numerous hitherto unexplained phenomena such as calmodulin kinase phosphorylation of MTs and its relation to actual memory storage in the human brain.

There are currently no data available in the experimental biophysics literature to verify the above numbers for memristance of MTs directly but work is underway to create a single MT trapping device, e.g. a microfluidic chamber or a nanochannel, to test these predictions. Below, we briefly discuss some preliminary experimental data we collected on ensembles of microtubules where pinched hysteresis loops were observed. While still preliminary, these observations are consistent with the theoretical predictions made in this paper.

## Experimental Measurements

We have previously reported impedance measurements for MTs and tubulin solutions under various conditions in Santelices *et al*.^32^ where details of the methodology used can be found. Such experiments, performing current-voltage measurements at both AC and DC voltages can potentially validate the memristive properties of MTs. Here, we report for the first time the observed hysteretic behavior of the buffer solution with ensembles of MTs.

**Figure 3:**
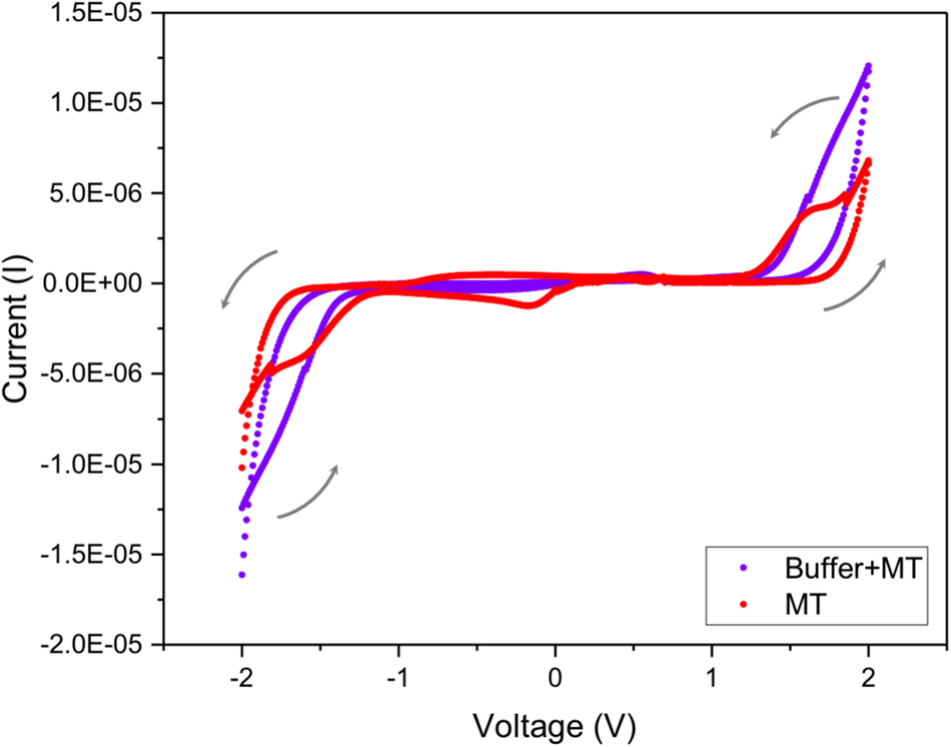
A characteristic pinched hysteresis loop for memristors. The purple data points show the observed behavior of MTs in the buffer solution. The red data points show the same data but with the contribution of the buffer subtracted. Grey arrows indicate the direction of the voltage sweep. For details regarding the protocol used, see the Methods section.

Specifically, by reversing the voltage applied we were able to observe a characteristic pinched hysteresis loop for memristors, which is shown in Fig. 3. A combined effect is shown for MTs and the buffer in which they are solubilized and a net effect on MTs by themselves where we have subtracted the contribution of the buffer.

**Figure 4:**
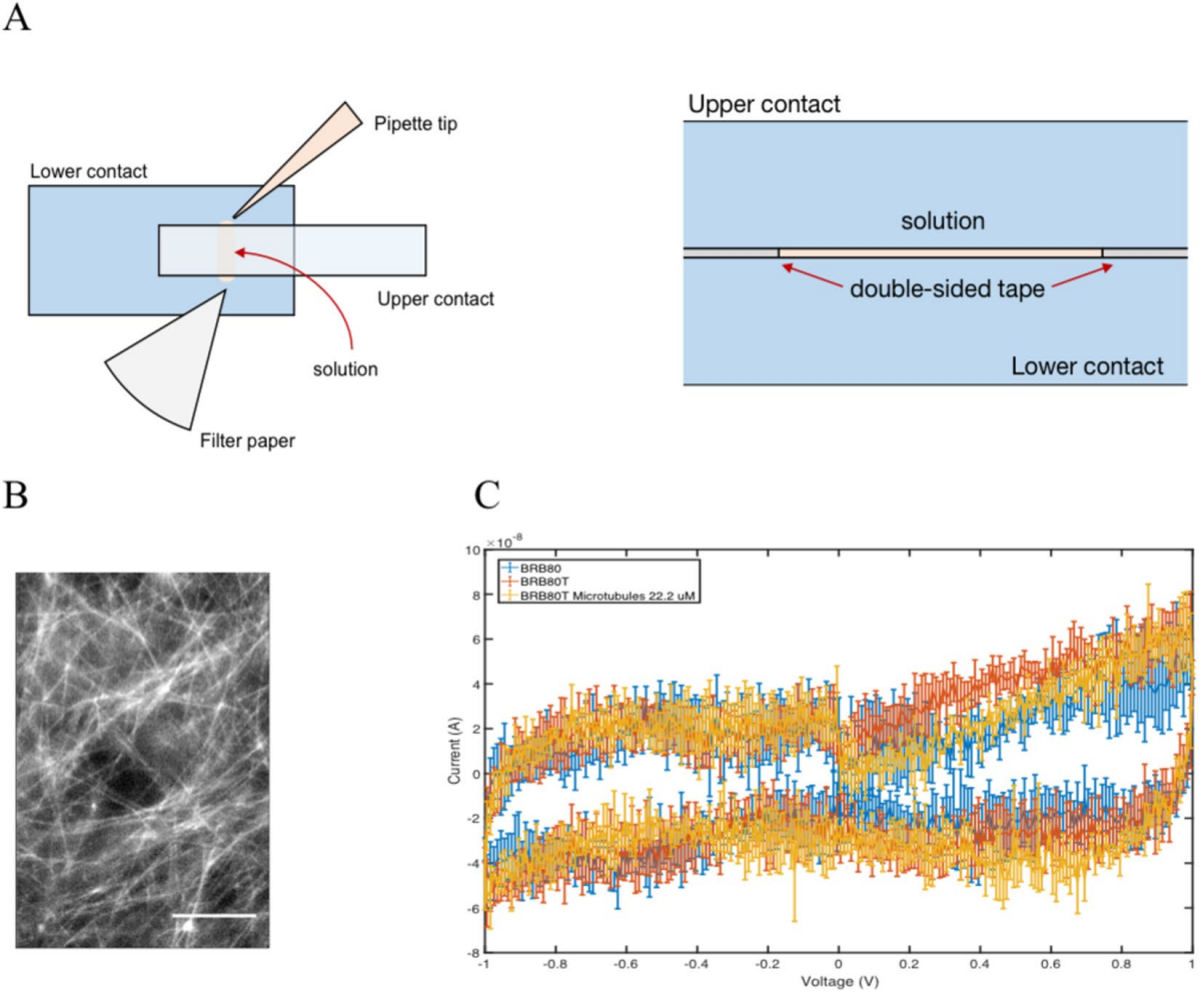
(**A**) A schematic displaying the top view (left) and the side view (right) of the parallel-plate device used to determine the electrical properties of MT solutions in the presence of BRB80 buffer with paclitaxel (BRB80T). (**B**) MTs imaged at 22 µM tubulin concentration using an epi-fluorescence microscope. Scale bar represents 100 μm. (**C**) Examples of current-voltage sweeps performed at 100 mV/s for BRB80T and BRB80T containing MTs at a 22 µM tubulin concentration. Error-bars represent standard deviations. Data were collected and averaged from three to five experimental sweeps. In spite of the large amount of noise, which is expected in such complex and irregular MT meshworks, (**C**) is indicative of a superposition of numerous pinched hysteresis loops providing support for the theoretical predictions in the previous sections of this paper.

Finally, we performed preliminary current-voltage measurements in a set-up shown in Fig. 4A using MTs at physiologically relevant concentrations of tubulin (22 μM)^41–43^ and in the presence of the BRB80T buffer (80 mM ionic strength). When we imaged MTs in such solutions using an epifluorescence microscope, we noticed that MTs formed unaligned and complex meshworks, resulting in a non-trivial bioelectric network (Fig. 4B). The high number of unaligned MTs results in a complex pattern of behavior that will be investigated in detail elsewhere. Nonetheless, current-voltage traces obtained through these measurements displayed complex hysteretic behavior (Fig. 4C).

## Conclusions

**Figure 5:**
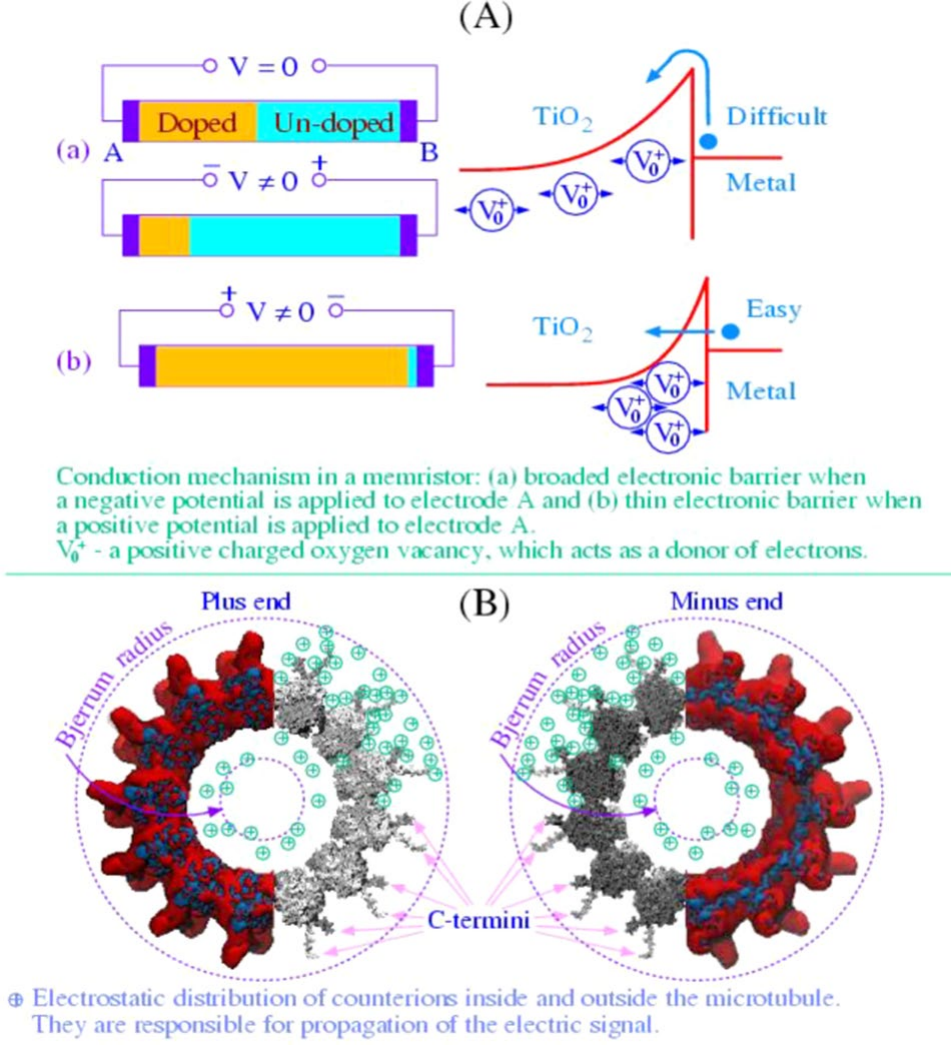
Comparison of the TiO_2_ memristor^3^ and the MT memristor shown from the plus end and from the minus end, respectively^15^: (**A**) positive charged oxygen vacancies play a role of memory carriers; (**B**) like role is played by counterions within the Bjerrum region adjoining to the MT from the outer and inner surfaces.

In this paper we have provided theoretical and experimental evidence in support of the hypothesis that MTs are subcellular memristors. MTs have highly negative linear charge densities, which are screened by counterions surrounding MTs from both outer and inner surfaces. The same counterions anchored by negative charges within the Bjerrum regions stay in fixed states until injected currents or potential gradients push them away from the MT vicinity. Ionic motion is guided by MT geometry but there are numerous intricacies due to the presence of nanopores on the MT surface, which enable ionic motion in and out of the lumen. Moreover, the highly-charged C-termini decorate the MT surface in a periodic pattern and can fluctuate between at least two conformational states: outstretched and bound to the MT surface. Transitions between these two states are susceptible to local electrostatic potentials and hence interact with ionic flows. We believe that these conformational transitions are responsible for memristive properties of MTs. In Fig. 5 we show an illustrative comparison between a TiO_2_ memristor where oxygen vacancies play the role of memory carriers and an MT memristor where ionic species are memory carriers.

**Figure 6:**
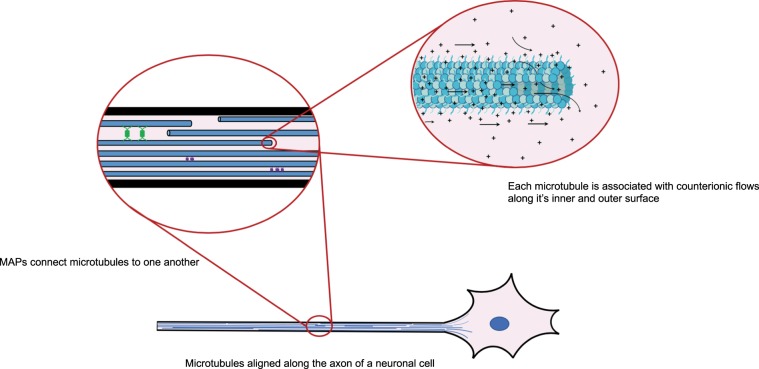
MTs form a complex ionic circuit within a neuron. A schematic displaying the role of MTs as individual circuit elements that transfer signals along a neuron. MAPs link MTs, enhancing long-range ionic transport across the neuronal cytoskeleton.

Overall our findings in the above experiments and simulations seem to indicate that MTs can propagate, and amplify, electric signals via ionic flows along the MT surface, through the lumen and across their nanopores. Simulations suggest that these flows are sensitive to the dynamics of the C-terminal region, and consequently are tubulin isotype-dependent since various tubulin isotypes are characterized, among other properties, by C-termini differences. So far there seems to be no direct experimental verification of role of the MT cytoskeleton in electrical signal conduction in neurons. However, there is significant indirect experimental evidence in support of MT’s being involved in human cognition and hence potentially in neuronal signaling. In Fig. 6 we show the distribution of MTs within the axons of neurons, which is intended to visualize how signals carried by ionic flows along MTs can be incorporated into the functions of neurons by interactions with MAPs, which can then be coupled to axoplasmic transport and can affect ion channels, for example.

The neuronal cytoskeleton has been purported to play a crucial role in learning processes and memory formation, which has been documented and reviewed^21,22,38^. Most eukaryotic cells exhibit MT dynamic instability with periods of growth interspersed with catastrophes and rescue events. However, MTs in neurons are less dynamic and more stable due to their interconnections with MAPs. However, reorganization of the MTs and MAPs in the neuronal cytoskeleton is known to occur during learning, which has been seen to correlate with an increase in MT numbers, and has also been shown to be impaired by the MT depolymerizing agent colchicine. This appears to indicate that learning involves dynamic MTs^21,44^. Work on the molecular basis of memory has implicated CaMKII (calcium/calmodulin-dependent protein kinase II) as crucial to LTP (long-term potentiation) contributing to learning and the memory formation^45,46^. CaMKII also phosphorylates both α- and β-tubulin directly in the C-terminal region of the protein^47^. An atomic-resolution model of MT phosphorylation by CaMKII^18^ demonstrates an intricate and potentially massive molecular code of information encryption in the structure of neuronal MTs, especially in dendrites, and this can be directly linked to current flows, which we argue possess memristive properties along MTs. Enzymatic reactions of this type may trigger MT matrix reorganization, which is required for memory formation and learning. A mechanistic understanding of memory encoding at the subcellular level now emerges, which is not only dynamic but inherently linked to subtle conductive properties of MTs, especially their memristive ionic conduction characteristics as argued in this paper^48^. There are several clear advantages offered by MTs as subcellular memristors. Tubulin is one of the most abundant proteins in neurons, and MTs are exceptionally well-conserved, spatio-temporally ubiquitous proteins. This suggests a widespread nature of MT memristors within the cell. Additionally, post-translational modifications (PTMs) on C-termini tails of tubulin that vary depending on the local and global MT environment may lead to complex attenuations in memristive action, depending on the positioning of MTs within the cell and on the cell type. The presence of MAPs provides further advantages and complexity to MT networks, creating connections among adjacent MTs (see Fig. 6) and establishing contacts between MTs and various macromolecules. While it is well-known that proteins degrade and denature over time, which would affect the endurance of MT-based memristors, some of it may be mitigated by stabilizing MTs via MAPs and pharmacological agents such as taxol. On the other hand, limited durability of MT-based devices offers new avenues such as the ability to construct evolvable bio-electronic devices or biodegradable or self-destructing ones.

Our discovery that MTs are biological memristors could, for example, help resolve the heretofore unknown origin of the impressive memory capabilities exhibited by the amoeba, which do not have neurons, let alone a brain, but are loaded with MTs. In general, MTs may offer numerous advantages over silicon-based technology. Microtubules are biological, biodegradable materials and hence offer an environmental advantage over seminconducting materials. They are very abundant in all eukaryotic organisms and highly conserved through evolution indicating their importance to living systems. Due to the diversity of C-termini sequences, which are cell-type and species-specific, there is a huge potential for designing an array of MT-based memristors with functional differences. This can be further amplified by post-translational modifications. Finally, MTs can form bioelectric circuits through their natural connections to MAPs, hence an enormous spectrum of circuit geometries is possible to be created even by self-organization processes.

## Methods

We performed I-V measurements on samples of MTs in buffer solution using a semiconductor characterization system (Keithley 4200-SCS) with a probe station. For this purpose, we created two-terminal and four-terminal electrical devices, made of Pt wires attached to a glass substrate in a flow cell, to test electrical changes due to MTs in physiological-like solution.

The electrical devices were constructed on a 10 cm square wafer. Each device, called EDA, has five wires, with contact pads large enough to be attached with probe tips to the Keithley 4200 semiconductor characterization system. The region where the wires converge has the five wires extending for 500 µm where the MTs can cross all 5 wires. In one device, the wires were fabricated to be 4 µm wide, with a 6 µm-space between wires (10 µm apart center-to-center). By visual inspection of fluorescent images, there appear to be 2, 3, 10, and 7 MTs making solid connections between wires 1 and 2, 2 and 3, 3 and 4, and 4 and 5, respectively.

MT polymerization was performed by first reconstituting tubulin powder (Cytoskeleton Inc, tl590m) according to the protocol provided by the supplier. The solution was subsequently snap-frozen in experimental sized aliquots. For each experiment, MTs were polymerized by incubating a tubulin aliquot (with a 45.45 μM concentration) at 37 °C for 30 minutes. BRB80 (80 mM PIPES pH 6.9, 2 mM MgCl_2_ and 0.5 mM EGTA (Cytoskeleton, Inc. BST01) supplemented with paclitaxel was added to this solution to attain the required tubulin and ionic concentration. To attain a final concentration of MTs at 22 μM tubulin, equal volumes of this solution and tubulin solution were mixed.

The flow cell was flushed with BRB4 buffer solution. Next, three sweeps of frequency sweeps for conductance measurements were performed. The flow cell was subsequently flushed with MT-containing buffer solution BRB4-MT1x after which three more frequency sweeps were implemented. An identical protocol was used for the following compositions of MTs and tubulin: BRB4-MT2x, BRB4-MT5x, BRB4-T1x, and BRB4-T5x, respectively. The buffer solution BRB4 was generated by diluting BRB80 20-fold with Milli-Q water. An HM Digital COM-100 EC/TDS/temperature meter was then inserted into the BRB4 buffer solution and the temperature was determined. After an incubation of one minute, the conductivity of each solution was measured and recorded.

To investigate the conductance of MTs and tubulin, we used diluted low ionic solution to lower the amount of ionic contribution to the overall conductivity. As usual, BRB4 ionic strength buffer was prepared by adding 5 μL of BRB80 buffer (80 mM PIPES pH 6.9, 2 mM MgCl_2_ and 0.5 mM EGTA (obtained from Cytoskeleton, Inc. BST01) to 95 μL Milli-Q water. BRB4-MT1x solutions (42.4 nM tubulin concentration) for testing were prepared by adding 5 μL of MT solution (850 nM tubulin concentration in BRB80) to 95 μL Milli-Q water. BRB4-MT2x solutions (84.8 nM tubulin) were prepared by adding 5 μL of MT2x solution (4 mM tubulin concentration in BRB80) to 95 μL Milli-Q water. BRB4-MT5x (212 nM tubulin) was prepared by adding 5 μL of MT5x solution (10 mM tubulin concentration in BRB80) to 95 μL Milli-Q water.

The range of applied voltages was ±1 *V* with 0.2 *V* step, from −1 *V* to 1 *V*. Linear regression was applied on the −0.6 to 0 *V* points to report a conductance. The slope calculated from linear regression is the sample’s conductance, and the inverse is its resistance. Multiple voltage sweeps were performed in each experimental situation. Signatone tungsten probe tips were used. Four-point collinear probe measurements were performed on the first four wires from the left of the EDA device, with a 1 nA applied current (the current range recommended by Keithley to give a voltage drop of about 10 mV) between wires 1 and 4. Wires 2 and 3 both measure the voltage respective to ground wire 4. Mean voltages are reported with an SD error. The current source range to determine the input impedance of wires 2 and 3 was set to be in the 1 nA range. The Keithley settings used were as follows: sampling mode, normal speed, interval 0.25, hold time 1. Ten measurements were performed with ~0.3 s between measurements, per execution.

For the experiments at physiologically relevant ionic strengths, a parallel-plate capacitor geometry composed of FTO (Fluorine-doped Tin Oxide) contacts was used, as displayed in Fig. 4A and described elsewhere (A. Kalra *et al*., unpublished, 2019). The distance between the plates was 70 μm and the total volume of the solution was 2.5 μL. These experiments were performed using a Zahner Zennium Impedance Analyzer in a two-probe configuration. Under these conditions there was no appreciable difference between the MT solutions and the controls. Experiments investigating the DC response of MTs at such physiologically relevant tubulin and ionic concentrations are presently under way.

## Supplementary information

Supplementary Information.

## Data Availability

The authors will provide the experimental data reported in this paper upon request.
