# Supplementary material for: Microtubules as Sub-Cellular Memristors
Source: Sci Rep. 2020 Feb 7;10:2108. doi: 10.1038/s41598-020-58820-y (PMC7005844; doi:10.1038/s41598-020-58820-y)
Supplement: Supplementary file 1 — Supplementary Information. [file 41598_2020_58820_MOESM1_ESM.pdf]

Supporting Information

Microtubules as Sub-Cellular Memristors

Jack A. Tuszynski[1,2,3], Douglas Friesen[1], Holly Freedman[4], Valery I. Sbitnev[5,6], Hyongsuk Kim[7], Iara Santelices[8], Aarat P. Kalra[2], Sahil D. Patel[8], Karthik Shankar[8] and Leon O. Chua[6]

[1] Department of Oncology, University of Alberta, Cross Cancer Institute, Edmonton, AB, Canada, T6G 1Z2

[2] Department of Physics, University of Alberta, Edmonton, AB, Canada, T6G 2E1

[3] DIMEAS, Politecnico di Torino, 10129 Turin, Italy

[4] Li Ka Shing Institute of Applied Virology, University of Alberta, Edmonton, AB, Canada, T6G 2E1

[5] St. Petersburg B. P. Konstantinov Nuclear Physics Institute, NRC Kurchatov Institute, Gatchina, Leningrad district 188350, Russian Federation

[6] Department of Electrical Engineering and Computer Sciences, University of California, Berkeley, Berkeley, CA 94720, USA

[7] Division of Electronics Engineering, Chonbuk National University, Jeonju, Jeonbuk, 561-756, South Korea

[8] Department of Electrical & Computer Engineering, University of Alberta, Edmonton, AB, Canada, T6G 1H9

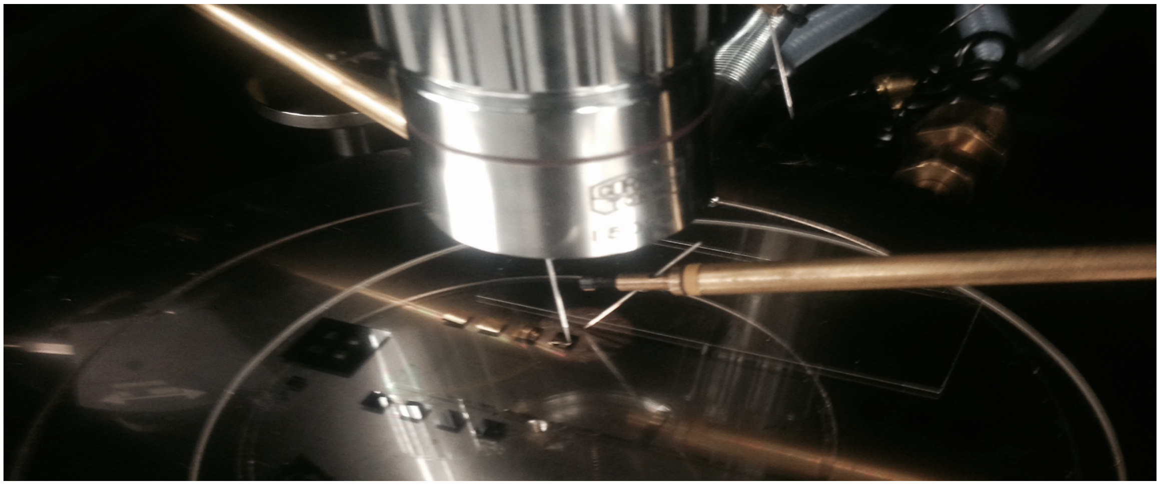

Figure S1. Experimental setup used to perform experiments using a two-probe geometry.

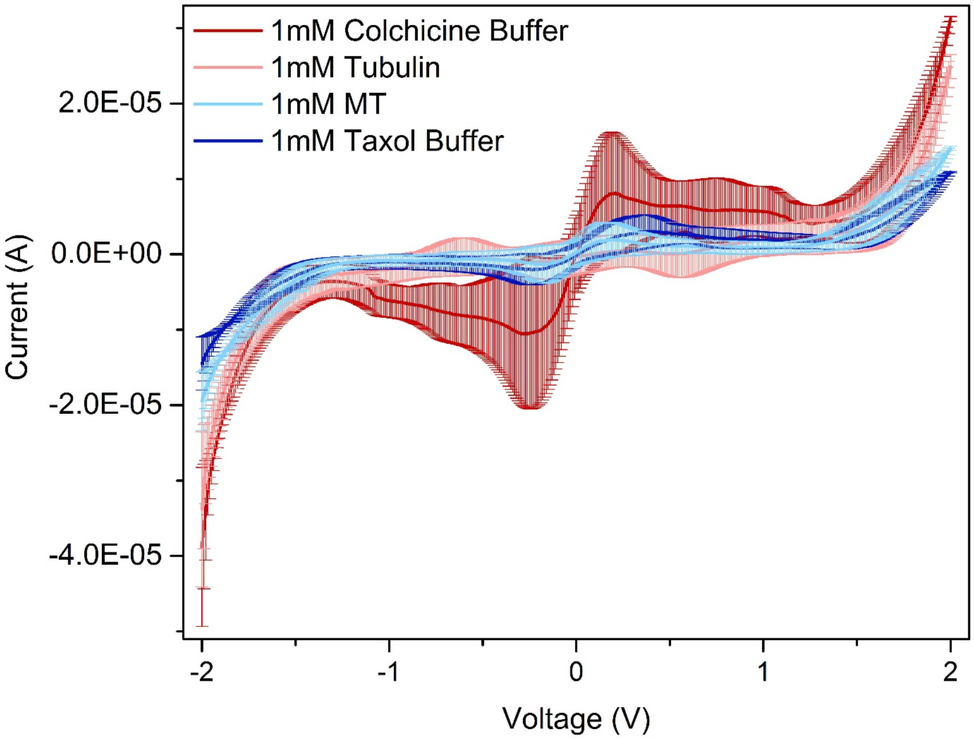

Figure S2. Summary plots of current-voltage trace of MT and tubulin solutions compared to their background solutions. 80 mM ionic strength buffer used

MT Aug 17 I-V

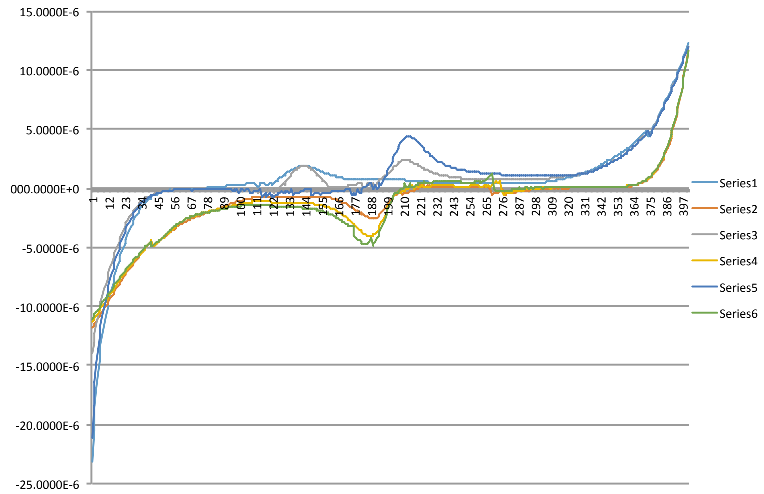

MT Aug 17 IV

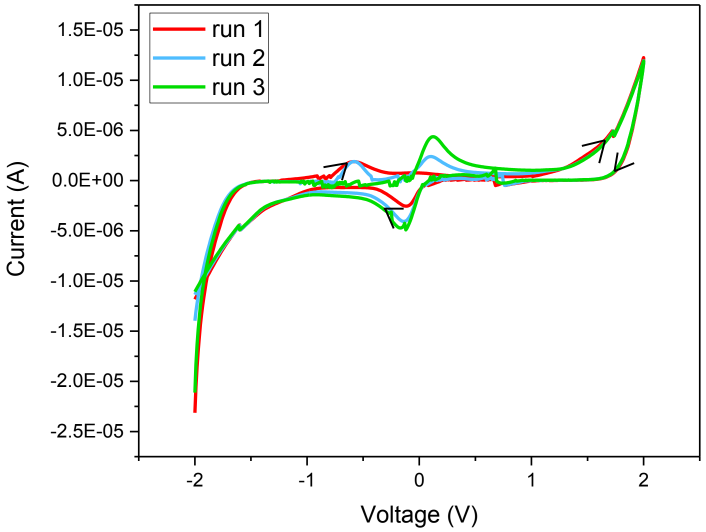

Figure S3. Examples of individual run plots of current-voltage trace for MTs in solution cwhere an 80 mM ionic strength buffer was used
